# Early Markers in Resistant Schizophrenia: Effect of the First Antipsychotic Drug

**DOI:** 10.3390/diagnostics12040803

**Published:** 2022-03-25

**Authors:** Georgi Panov Panov

**Affiliations:** Psychiatric Clinic, University Hospital for Active Treatment “Prof. D-r Stoian Kirkovic”, 6000 Stara Zagora, Bulgaria; gpanov@dir.bg; Tel.: +359-88-8454634

**Keywords:** resistant schizophrenia, antipsychotics, first antipsychotic drug, effect of therapy, treatment consideration, remission consensus schizophrenia

## Abstract

Background: Schizophrenia is a mental illness with a multifactorial etiology and clinical presentation. Treatment is mainly with antipsychotic drugs. Despite the increasing number of antipsychotic drugs, there has been no significant change in the percentage of resistant cases. These data gave us reason to look for a link between the effect of the first individually selected antipsychotic drug and the established resistance to therapy. Method: An assessment has been made of 105 patients with chronic schizophrenia with consecutive psychotic episodes. The choice of antipsychotic has been made on the basis of clinical features, history of efficacy of previously used neuroleptics, anthropometric features, as well as somatic comorbidities. Accidental use of benzodiazepines in anxiety conditions as well as correctors in indications for extrapyramidal problems have been reported. Assessment was made based on clinical observation as well as on changes in PANSS score. Results: Of the 105 observed patients, the effectiveness of the first antipsychotic effect was found in 46.7% of patients. Follow-up of patients for a period of 12 weeks revealed that 45 (42.8%) of them had resistant schizophrenia, while the remaining 60 (57.2%) achieved clinical remission and initial functional recovery. The effect of the first antipsychotic drug was established in 9 (20%) of the patients with resistant schizophrenia and in 40 (66.57%) of the patients in clinical remission. Conclusion: The evaluation of the first antipsychotic medication is significant for the prognosis of patients with schizophrenia. Its lack of effectiveness indicates a high probability of resistance and can be a good indicator of earlier change and a possible search for more “aggressive” measures to prevent future resistance and possible disability.

## 1. Background

Diagnostic categories are constructions that change over time as a consequence of research and scientific achievements in the field. In the domain of psychiatry, there are accelerated processes of change and redefinition of nosological constructions related to attempts to connect the psyche, consciousness, and brain [1]. There is a term in Latin “*diagnosis ex juvantibus*”—the diagnosis is made based on the effect of treatment. Can it be redefined to “*prognosis ex juvantibus*“, meaning that the prognosis can be made on the effect of treatment?

Schizophrenia is a mental illness characterized by psychotic symptoms: delusions and hallucinations, as well as cognitive symptoms. It is often accompanied by anxious, affective, and obsessive symptoms [2]. The idea of schizophrenia as only a mental illness differs from modern research, which revealed the presence of complex disturbances in metabolic and immunological aspects, lipid profile, and severe changes in the opioid system [3,4]. These combined disorders in psychiatric and metabolic aspects give grounds to look for a comprehensive approach to their treatment [5,6]. Impaired cognitive processing of information about both reality and self-perception leads to changes in behavior, metabolism, and functional disturbances in neural networks and connections between brain centers and neural populations [7,8,9,10,11,12,13,14]. Complex metabolic changes in schizophrenia are also supported by studies using various psychotropic drugs. They show that in addition to their central effect, they also affect the metabolism of patients, and different drugs have a different influence on the metabolic profile [6]. Of course, the question arises whether the use of different drugs with different receptor profiles would meet the individual needs, correcting not only the central dopamine disbalance but also the associative lipid and immunological parameters. These complex and serious changes in patients with schizophrenia are associated with a relatively high prevalence of individuals at the state of a clinical high risk of psychosis in the main population—1.7%, and in clinical groups, this percentage reaches 17% [15]. The complex combined disturbances in patients with schizophrenia are the reason why, despite the constant expansion of various therapeutic interventions, a significant percentage of patients remain resistant and represent a serious personal family and social problem. This is why some authors try to consider patients with resistant schizophrenia as a separate category. This raises the question of seeking different therapeutic approaches [5,16]. The fact that different drugs have not only different receptor profiles of impact but also different effects on metabolism [6] raises the issue that individual approaches in the treatment of patients should be sought. In addition, different drugs have different effects on the work of neuronal populations. This effect gives reason to assume that with prolonged use, there will be a change in the neuronal substrate caused by them [17,18].

One of the most challenging questions in the treatment of schizophrenia is deciding how to evaluate the effect of the first antipsychotic medication, how its effect should be analyzed, and how long to wait before deciding to replace it with another one or start combination therapy. For decades, the effect of antipsychotic drugs has been considered to be delayed over time, and it has been recommended to re-evaluate the effect of treatment after 4–8 weeks [19]. Other researchers have found opposite results. They compared the effect of treatment at 48 h and 28 days. They found that early change in therapy is a predictor of disease prognosis [20]. In another study to assess the effect of treatment, researchers used more than a 20% reduction in the Brief Psychiatric Rating Scale (BPRS) points in the first week to consider patients responding early to antipsychotic therapy. They found that those who did not respond to therapy in the first week did not respond to the fourth week of treatment [21]. In a meta-analysis, it was found that a response may occur earlier in therapy and within the first one or two weeks [22,23]. These studies even demonstrate that the first week has the highest rate of reduction in psychotic symptoms (13.8%) and emphasize that the first week of treatment as a manifestation of effectiveness is particularly important, and long-term change in the BPRS scale at 4 weeks in these patients is equal to the change in the first year in those in whom no such early change was registered [22,23,24]. Comparing patients who did not have at least minimal improvement in symptoms after 2 weeks of treatment (“early and unresponsive”) with those who did, it was found that the latter had a better response to symptoms, improved functioning, and a higher degree of remission achieved [25,26].

Most studies of early response or lack of response have been conducted retrospectively and have shown that lack of response is a reliable predictor of subsequent ineffectiveness in continuing treatment with the same drug [25,26,27]. These studies also show that the majority of patients (almost 70%) do not meet this criterion for “early response” with either typical or atypical antipsychotic medications [26,28].

The clinical dilemma in those patients who do not show an early response is whether the patient should continue treatment with the same drug or make a substitution with another. Cases of favorable outcomes have also been reported in patients with poor or limited response to the first antipsychotic drug after switching to another [29,30,31]. These studies usually do not have a control group and the period during which they were on the first drug has not been evaluated, and therefore we cannot conclude that switching is indeed a useful option. Importantly, no benefit was reported from switching antipsychotic drugs in two clinical intervention efficacy trial (CATIE) analyses, which included a control group [32,33]. There are also opposite studies which show that when changing medications (two analyses from risperidone to olanzapine and vice versa), an effect was found in about 30% of patients after the change [34,35].

Another study in patients with chronic schizophrenia, and schizoaffective disorder (533 patients from different centers) showed that the first drug, risperidone, achieved a response of a 20% reduction in PANSS in 28% of all patients in the second week of the study. In the remaining 70%, such a response was not observed [36,37]. Given the fact that the study included various groups of patients in the analysis of only patients with schizophrenia, the recalculated response to risperidone is about 38% of them. In the study of Kinon, Chen, Ascher-Svanum et al. (2010), patients were selected without clear inclusion and exclusion criteria (only diagnosed) and only risperidone was used for the study (without taking into consideration the effectiveness of previously used drugs, as well as the need for treatment to be tailored to the individual characteristics of the patient) [37]. Naturally, the question arises as to why risperidone and whether the results would be the same when using another antipsychotic. On the other hand, these results do not reflect the real situation in psychiatric practice (clinics, wards, outpatient care), giving only one drug to all patients.

There is no single antipsychotic that is paneffective, as individuals’ responses and tolerance to individual medications vary widely [38]. Other studies also indicate that different drugs have different therapeutic profiles and different side effects, which requires an individual approach to their use [39]. An analysis of 1500 patients showed that 74% stopped their treatment due to a lack of effect or due to side effects, which emphasizes the need for an individual approach to treatment [40].

In practice, the choice of drug is based on the clinical setting, concomitant comorbidity consistent with the profile of the antipsychotic used, medical history, and retrospective evaluation of effectiveness in the use of previous drug regimens and individual tolerance to treatment.

Regarding the significance of the effect of the first two weeks of treatment, there are opposite results. A study of 20 patients with schizophrenia with a much shorter duration of the disease (up to 5 years) showed that the effect of treatment in the first 2 weeks of therapy does not give an idea of the overall effectiveness of therapy compared to 12 weeks [41]. The authors point out that the results of previous studies have been conducted in patients with chronic schizophrenia and this may not be indicative in assessing the effectiveness at the beginning of therapy. On the other hand, in patients with the first psychotic episode, the efficacy analysis also cannot be accurately determined with respect to the development of future resistance due to the fact that the resistance cases after the first psychotic episode are about 10–15% [42]. These data indicate that in most cases, resistance develops later in therapy and as a process of disease progression.

The above shows that with regard to antipsychotic drugs, there are mixed data on their effectiveness, the time of manifestation of the effect, and in terms of the effectiveness of changing therapy. We have not found a study that allows the use of the therapeutic effect as a diagnostic method. Such an analysis has been carried out in the field of epilepsy [43].

Based on these facts, we tested the following hypothesis in a perspective way: the effect of the first individually selected antipsychotic drug will be crucial for the prognosis of future resistance.

## 2. Material and Methods

A total of 105 patients with schizophrenia have been observed. An assessment has been made of patients with chronic schizophrenia with a consecutive psychotic episode. Of these, 45 have resistant schizophrenia and the remaining 60 are in clinical remission.

The type of study is correlation analysis.

Prospective evaluation of the patients has been performed.

The diagnosis has been made according to the criteria of ICD 10 and DSM 5 [44].

Patients with the first psychotic episode were not included in the study due to the low rate of resistance and that they would not be representative for assessing resistance in patients with schizophrenia in general [42].

Inclusion criteria for patients with resistant schizophrenia were those who have met the resistance criteria of the published consensus on resistant schizophrenia [45], which are:

1. Assessment of symptoms with the PANSS (The Positive and Negative Syndrome Scale) and BPRS (Brief Psychiatric Rating Scale) [24,36].
2. Prospective monitoring for a period of at least 12 weeks.
3. Administration of at least two antipsychotic medication trials at a dose corresponding to or greater than 600 mg chlorpromazine equivalents.
4. Reduction of symptoms when assessed with the PANSS and BPRS by less than 20% for the observed period of time.
5. The assessment of social dysfunction using the SOFAS (Social and Occupational Functioning Assessment Scale) is below 60 [46].

The exclusion criteria were:

1. Mental retardation.
2. Presence of organic brain damage.
3. Concomitant progressive neurological or severe somatic diseases.
4. Expressed personality change.
5. Score of MMSI below 25 points.

The use of antipsychotic drugs as a first-line treatment and based on data about previous efficacy and tolerability, and considering the fact that this was a consecutive episode of psychosis that required treatment, we used haloperidol, risperidone, olanzapine, and amisulpride.

Rigorous clinical monitoring was performed to determine a change in general condition in order to assess whether to make an early change in the medication strategy in case of insufficient efficacy or in the presence of intolerable side effects. As a method of objective assessment, the PANSS was used to register the change in mental state.

The use of benzodiazepine/hypnotics/anxiolytics was permitted during the study, but only for the treatment of anxiety or insomnia as clinically indicated. Patients receiving a stable dose of antidepressants, anticonvulsants used as a mood stabilizer, or lithium therapy for at least 30 days before study initiation could continue on these concomitant medications at a stable dose. However, the doses of these medications could not be changed in an attempt to enhance efficacy.

The statistical software package SPSS was used for statistical data processing. Since we use nominal variables, and limit group numbers, the chi-square test of non-parametric tests was chosen for comparing the groups, and the data were analyzed.

## 3. Results

The mean age of patients in the group of resistant schizophrenia was 36.98 years, the minimum being 21 years and the maximum 60 years.

The mean age of patients in the group of schizophrenia in clinical remission was 37.25 years, with a minimum of 23 years and a maximum of 63 years.

We did not find a difference in the mean age of the patients in both groups at the time of the study.

**Figure 1:**
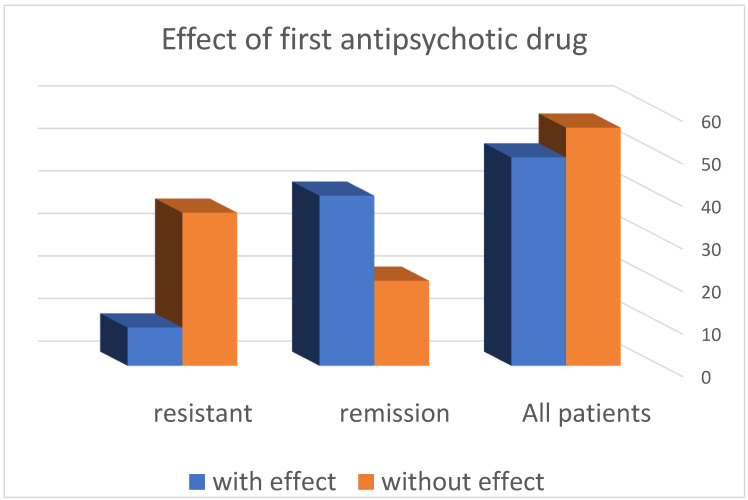
Distribution of patients according to the effect of treatment.

Efficacy of the first antipsychotic drug selected individually for the individual patient was consistent with the symptom profile and previous history of the effect of one or another antipsychotic effect. The profile of side effects, tolerance to dose ranges, and the presence of concomitant somatic diseases requiring a particular therapy were taken into account. For all patients observed by us, both those with resistance and those in clinical remission, the results are presented in Table 1 and Figure 1.

We found a response after administration of 1 antipsychotic drug in 49 (46.67%) of the patients, while there was lack of effect in the remaining 56 (53.13%). From this distribution, it is clear that the probability of achieving effectiveness with the first antipsychotic drug is almost like a game of “heads or tails”, i.e., 50/50.

- The distribution of patients according to the response to the first antipsychotic drug in the resistance group shows that in 36 (80%) patients, no effect was observed, and in the remaining 9 (20%), such an effect was registered.
- In the group of patients in remission, it was found that in 20 (33.33%) patients, no effect was observed when using the first neuroleptic, and in the remaining 40 (66.57%), an effect was found.
- These results show that patients with resistant schizophrenia are 2.5 times more likely to have no effect after administration of the first antipsychotic drug compared to patients in remission (*** *p* < 0.001) (Table 2 and Table 3).

## 4. Discussion

This result can be considered in the context of the observations of other authors, namely that the lack of effect of one drug in an adequate dose is often associated with the lack of effect of other drug regimens that will be administered later [47]. These analyses provide a reason to emphasize why this indicator, namely the “effect of the first antipsychotic drug” (individually selected), is important as an indicator for predicting the development of the schizophrenic process.

We have found that the effect of the first antipsychotic drug is of particular importance in the analysis of possible future resistance in patients with schizophrenia. We have found an interesting coincidence of our results with the data of other authors in the analysis of patients with epilepsy. They found that the effect of the first antiepileptic drug was registered in 47% of patients [43]. We find this coincidence extremely interesting given the fact of biological antagonism in the two diseases—epilepsy and psychosis [48].

Arguments can be made that the effectiveness of the first drug is not indicative of the fact that some patients show a delayed response [49]. In these cases, the drug is often replaced with another antipsychotic medication. We accept the presence of such cases even more so considering that a significant percentage of our patients in the group who achieved remission failed treatment with the first antipsychotic drug (Table 1). Other analyses have shown that the success rate of treatment with a second antipsychotic medication (except clozapine) after initial failure with the first one is less than 20% [50]. This fact, in our opinion, again emphasizes the need for the right choice of antipsychotic drugs in the treatment of patients with schizophrenia. Most studies have shown that the effectiveness of treatment in the first weeks of therapy is indicative of the development of future resistance. On the one hand, it is found that the highest degree of effectiveness in the presence of such resistance is observed in the first week of therapy [21,22,23]. On the other hand, there is a different point of view. There are studies that show that different antipsychotic drugs have different times before their antipsychotic effect begins [51]. In this sense, it is necessary to wait a different period of time for different medications to appear effective. After this period, it may be considered whether to change the medication or increase the dose. We see this as a reason not to use the same timeframe for different medications. It should be kept in mind that in clinical practice, it is rare to wait several weeks in search of efficacy in the use of individual drugs. There is another point of view that must be taken into account in addition to the difference in effectiveness between drugs. Different antipsychotic drugs have different profiles of side effects, and in this respect, it is inappropriate to argue that their effectiveness would not be the same in a particular patient. This is the reason why we use different medications [52,53]. The various side effects of individual medications are also related to the search for an individual approach and method of administration of antipsychotic medications in the individual patient [40,53]. Side effects are the reason why many drugs, despite the good therapeutic effect, are withdrawn from clinical practice [54].

We can conclude that the analysis of only one drug administered to a large number of patients is not able to give us the necessary information about the effectiveness and have the necessary informative value for future resistance, i.e., in many cases, this will not be the most appropriate medication. This is also the most probable reason for the differences we found between our results and the analysis of Kinon, Chen, Ascher-Svanum et al. (2010) [37]. On the other hand, the individual response to antipsychotic drugs varies widely, and there is no single drug that is paneffective [38]. We found in 20% of resistant patients an early effect of treatment that was not maintained over time. Studies show that despite taking antipsychotics, relapses occur in about 27% of patients [55]. 

The established lack of sufficient effectiveness of the use of various antipsychotic drugs has made it possible to seek other non-invasive strategies for the treatment of individual mental disorders. Such a possibility is the use of transcranial magnetic stimulation (TMS). In mental disorders, a number of combined disorders in neuronal activity have been identified, associated with both changes in neurotransmission and the activity of various neuropeptides [56]. The remodeling of neuronal activity or neural networks as well as the change in neuropeptide activity is an effect that has been found in the use of transcranial magnetic stimulation [57,58]. Due to the relatively new use of TMS in clinical practice, there are no comparative studies to evaluate the effectiveness of TMS over drug therapy.

The effectiveness of treatment is a big problem not only in the field of psychiatry, but also in other medical specialties. The main difference between the efficacy studies conducted in patients with schizophrenia is mainly limited to evaluating a particular drug or at a certain time interval without analyzing the individual characteristics of patients. We apply treatment based on the individual characteristics of the patient by analyzing the response to the chosen antipsychotic drug. Our study shows that even during other diagnostic procedures, the effect of the first antipsychotic drug is able to give us good direction for the prognosis of patients with schizophrenia.

One of the limitations of our study is the number of patients. We believe that the study can be repeated, but with a much larger number of patients from different centers. This would provide more accurate information on the use of the “first antipsychotic effect” criterion as an assessment of the prognosis. On the other hand, this assessment will always raise the question of whether this is the most accurate medicine used in each case. We believe that even if we fail to find the right medicine for a patient, we will in principle be able to build a general picture of the effectiveness of this criterion.

## 5. Conclusions

The evaluation of the first antipsychotic medication is crucial for the prognosis of patients with schizophrenia. Often in clinical practice, we do not pay enough attention to the effect of the first drug used, making frequent changes in therapy, and later in the course of the overall treatment, we realize that the patient has resistant schizophrenia. We found that patients with resistant schizophrenia are 2.5 times more likely to have no effect after the first antipsychotic drug than patients in remission. The lack of effectiveness indicates a high probability of resistance and can be a good indicator of earlier change in therapy and a possible search for more “aggressive” measures to prevent future resistance and possible disability. Additional augmentation strategies such as mood stabilizers or earlier treatment with clozapine, as well as early consideration of electroconvulsive therapy, would be valuable in the overall assessment of the individual case.

## Figures and Tables

**Table 1:** Distribution of patients according to the effect of treatment. Sch = schizophrenia.

First Antipsychotic Medication			
	Count	With Effect	Without Effect
All patients	105	49 (46.7%)	56 (53.3%)
Resistant Sch	45	9 (20%)	36 (80%)
Remission Sch	60	40 (66.57%)	20 (33.33%)

**Table 2:** Statistical significance of the results.

		Value	Asymptotic Standard Error	Approximate T	Approximate Significance
Interval-by-Interval	Pearson’s R	0.463	0.084	5.300	0.000
Ordinal-by-Ordinal	Spearman Correlation	0.463	0.084	5.300	0.000
No. of Valid Cases		105			

**Table 3:** Statistical significance of the results.

Chi-Square Tests					
	Value	df	Asymptotic Significance (2-Sided)	Exact Sig. (2-Sided)	Exact Sig. (1-Sided)
Pearson’s Chi-Square	22.500	1	0.000		
Continuity Correction	20.664	1	0.000		
Likelihood Ratio	23.676	1	0.000		
Fisher’s Exact Test				0.000	0.000
Linear-by-Linear	22.286	1	0.000		
No. of Valid Cases	105				

## Data Availability

The raw data supporting the conclusions of this article will be made available by the authors upon reasonable request.
